# Veterinary Students’ Perception and Understanding of Issues Surrounding the Slaughter of Animals According to the Rules of Halal: A Survey of Students from Four English Universities

**DOI:** 10.3390/ani9060293

**Published:** 2019-05-30

**Authors:** Awal Fuseini, Andrew Grist, Toby G. Knowles

**Affiliations:** School of Veterinary Science, University of Bristol, Langford, Bristol BS40 5DU, UK; andy.grist@bristol.ac.uk (A.G.); toby.knowles@bristol.ac.uk (T.G.K.)

**Keywords:** animal welfare, stunning, religious slaughter, veterinary students, Halal meat

## Abstract

**Simple Summary:**

Veterinarians play a vital role in safeguarding the welfare of animals and protecting public health during the slaughter of farm animals for human consumption. With the continued increase in the number of animals slaughtered for religious communities in the UK, this study evaluates the perception and level of understanding of religious slaughter issues by veterinary students at various stages of their studies. The results showed a significant effect of the year of study on respondents’ understanding of the regulations governing Halal slaughter and other related issues. Whilst the prevalence of vegetarianism and veganism were higher than the general UK population, the majority of respondents were meat eaters who indicated that they prefer meat from humanely slaughtered (pre-stunned) animals, irrespective of whether slaughter was performed with an Islamic prayer (Halal) or not.

**Abstract:**

The objective of this study was to evaluate the perception and level of understanding of religious slaughter issues, and the regulations governing the process, amongst veterinary students in England. A total of 459 veterinary students in different levels, or years of study (years 1–5), were surveyed. On whether there is a need for food animals to be stunned prior to slaughter, the majority of respondents 437 (95.2%) indicated that they would want all animals to be stunned before slaughter, including during religious slaughter, 17 (3.6%) either did not have an opinion or indicated ‘other’ as their preferred option and 5 (1.1%) indicated that religious slaughter should be exempt from stunning in order to comply with traditional religious values. The results showed a significant association between respondents’ year of study and (i) their understanding of UK animal welfare (at slaughter) regulations, (ii) their recognition of stunning as a pain-abolishing procedure and (iii) the likelihood of them wittingly purchasing and consuming meat from animals that have been stunned prior to slaughter, and also classified as Halal.

## 1. Introduction

The slaughter of food animals, whether stunned or not, is an emotive issue that has long divided opinion. Those against the production of farm animals, their slaughter and the subsequent consumption of meat have often cited the effect of livestock agriculture on the environment [1,2,3,4], a decline in the population of wild animals as a consequence of cultivating animal feed [5] or simply put a case for animal rights [6] or religiosity [7]. However, the perceived importance of meat in the diet of man cannot be underestimated. Meat is seen by many as an important source of proteins, amino acids, vitamins and other essential nutrients required for the sustenance of life [8,9]. The slaughter of animals in many industrialised economies is a highly regulated procedure; these regulatory measures are put in place to protect the welfare of animals (and the health and safety of operatives) and to ensure that meat is fit for human consumption. Within the European Union, the protection of animals at the time of slaughter is regulated under Council Regulation, EC1099/2009 [10] which specifies acceptable pre-slaughter procedures and approved slaughter methods for food animals. To protect animal welfare, EC1099/2009 requires the stunning of all animals prior to death (itself caused by bleeding out), with the exception of animals slaughtered in accordance with religious rites, this being mainly for Shechita and Halal slaughter. Halal slaughter is practised by followers of the Islamic faith, and animals are required to be alive prior to bleeding and a prayer is said by the slaughterer at the time of neck-cutting on every animal. Shechita slaughter, on the other hand, is practised by followers of the Jewish faith, and again animals are required to be alive and a prayer is said; however, during Shechita slaughter, there is no requirement for the prayer to be recited on every animal. Whilst some Muslims accept stunning during Halal slaughter, the Jewish community unanimously reject all forms of stunning. Member states can apply a derogation to permit slaughter without stunning, and this derogation is in place in the English domestic regulation, the Welfare of Animals at the Time of Killing (WATOK 2015) Regulation [11]. Research using, for example, Electroencephalogram (EEG) recordings of the electrical activity of brain has demonstrated that this method of slaughter does compromise animal welfare [12,13,14]. Contrary to the findings of Gibson and colleagues [12], other researchers [15] subjectively observed the behaviour of some three thousand cattle slaughtered without stunning (in line with the Shechita rules) and concluded that, ‘in their opinion’, the animals did not exhibit overt behaviours that were consistent with pain. The UK and other member states have used the derogation to permit slaughter without stunning under strict conditions. In Great Britain, the domestic regulation (WATOK) [11,16,17] requires animals to be individually and mechanically restrained during slaughter without stunning, and that ruminants must not be moved after the neck-cut until they lose sensibility. As a regulation, sheep must stand still for at least 20 s whilst cattle must not be moved for at least 30 s following the neck-cut. The essence of the standstill time is to ensure that animals lose sensibility due to blood loss before they are moved in order to avoid any additional pain or distress associated with the process. Further, abattoirs slaughtering animals without stunning must also have a backup stunner (a requirement also of abattoirs using pre-slaughter stunning) to be used in the event of delayed loss of consciousness after the neck-cut.

Animal rights and welfare groups continue to publicise the negative aspects of slaughter (both stun and nonstun) with a view to highlighting welfare compromises during slaughter. Over the last decade or so, UK-based animal welfare charity, Animal Aid, has released several covert recordings [18] taken in abattoirs that have highlighted animal suffering and have used this as an argument for veganism [19]. Harper and Makatouni [20] suggested that consumers are becoming well informed about the welfare aspects of livestock agriculture and are opting for welfare-friendly products. Some consumers are well informed about the role official veterinarians (OVs) play in safeguarding animal health and welfare. Wall [21] reported that the role of OVs is paramount in the implementation of the ‘one health’ initiative which is a collaborative, multidisciplinary approach to ensuring the optimal attainment of good health and welfare of human and nonhuman animals globally. OVs receive specialist veterinary training to be licenced to work in abattoirs to safeguard animal welfare and human health. However, Spinka [22] noted that there are gaps in the level of knowledge of OVs across the EU due to differences in the modules taught and the depth of subjects covered by different EU universities.

As potential future enforcers of religious slaughter laws in the UK (and other parts of the world), veterinary students at four English universities were recruited to participate in this study. The aim was to evaluate their perception and understanding of the regulations governing religious slaughter as it stands in the UK. The paper further examines the difference in the level of understanding of these issues amongst different year groups. To the best of our knowledge, there are no prior published data on veterinary students’ perception and understanding of religious slaughter.

## 2. Materials and Methods

### 2.1. Data Collection and Sampling Methods

A total of 459 veterinary students from four universities in England participated in the study; University of Bristol (*n* = 344), University of Nottingham (*n* = 57), University of Liverpool (*n* = 45) and Royal Veterinary College (*n* = 13). Prior to the survey, all students were provided information on the aims and objectives of the study and all respondents gave informed consent to participate in the study. Respondents’ data were anonymised. Data were collected using ‘SurveyMonkey’ online software by sending a weblink to students to allow them to participate at a time convenient to themselves. One of the possible limitations of this study is that respondents were not asked about their religion. The University of Bristol’s Ethical Review Board granted ethical approval for the study (ID75001).

### 2.2. Data Analysis

Responses to questions are reported as percentages with actual number of respondents contributing following in brackets. Exact chi square tests were used to test for associations between categorical variables; where there were ordered categorical variables, a test for trend was carried out using an exact Gamma statistic (IBM SPSS Statistics Version 25, IBM, Inc., New York, NY, USA).

## 3. Results

One respondent was dropped from the analysis because he/she did not answer a majority of the questions. Elsewhere, where there were occasional missed questions; those respondents are not included in the count or calculation of percentage. Omissions are treated as missing at random (there were 12 questions requiring a response, and there were 8 individual (respondents) omissions in total across 7 of these questions). The mean age of respondents was 22 with a range of 18–39. Respondents were recruited from four universities in England offering veterinary degrees, and the majority of respondents indicated their programmes of study as veterinary science 98.9% (454), with the remaining 1.1% (5) selecting ‘other’ (Veterinary Medicine and Surgery, Veterinary Medicine, Bioveterinary science and Veterinary Medicine with Intercalation) as their programmes of study. The levels or years of study of respondents were 22.9% (105) in the fourth year, 22.7% (104) in the third year, 21.2% (97) in the second year, 18.3% (84) in the fifth year and 14.9% (68) in the first year. Respondents’ homes of origin were from cities and towns across the UK with the larger proportions of respondents indicating they came from London 6.1% (28), Bristol 6.1% (28), Nottingham 2.8% (13) and Manchester 2.8% (13) areas.

Respondents were asked whether they were meat eaters and if so, their level of meat consumption. One respondent did not answer this question. The majority of respondents indicated that they were meat eaters, 81.3% (372), whilst 19.2% (88) indicated that they did not eat meat and 0.9% (4) chose only dietary exclusions by choice. Of the 372 meat eaters, 62.5% (286) indicated that they ate meat regularly, whilst 18.8% (86) said they ate meat occasionally. The 88 respondents who indicated that they did not eat meat were 15.3% (70) vegetarians and 3.9% (18) vegans. Respondents were presented with the statement: ‘At slaughter, the death of an animal takes place because the major blood vessels are severed, and critical blood loss occurs. This process is thought to be painful’ and were then asked if they agreed with a series of statements. Two respondents did not answer these questions. The majority of respondents, 90.4% (413) indicated that ‘pre-slaughter stunning abolishes the pain associated with the neck-cut during slaughter’, whilst 9.6% (44) selected the option ‘pre-slaughter stunning cannot abolish the pain associated with the neck-cut during slaughter’. There was a significant association between respondents’ year of study and their response to whether stunning is capable of abolishing the pain associated with the neck-cut (chi sq. = 33.0, df = 4, *p* < 0.001). The proportion of those agreeing that pre-slaughter stunning abolishes the pain associated with the neck-cut during slaughter were 79.4% (54), 80% (77), 95.2% (99), 98.1% (103) and 95.2% (80) of years 1, 2, 3, 4 and 5, respectively (proportions per year). The perception and understanding of respondents with regard to halal slaughter in the UK was evaluated (one respondent did not answer this question). The majority of respondents, 36.9% (169) selected the following option: ‘the majority of animals are not stunned, but some Muslims will accept meat from stunned animals as being Halal’, 29% (133) selected ‘all animals are required to be slaughtered without stunning’, 23.1% (106) selected ‘the majority of animals are stunned as most Muslims accept meat from stunned animals as Halal’, whilst 10.9% (50) respondents indicated that they were not sure about the situation of Halal slaughter in the UK. The results indicated a significant association between the year of study and respondents’ understanding regarding the situation with Halal slaughter in the UK; in later years of study, students understanding tended to improve (chi sq. = 84.2, df = 12, *p* < 0.001). Respondents’ awareness about the permissibility of slaughter without stunning for religious slaughter was evaluated (see Table 1). One respondent did not answer the question, 90.4% (414) indicated ‘Yes’, whilst 9.6% (44) indicated ‘No’. There was a significant association between respondents’ year of study and their response to the above question, with a trend across years (a trend of increased awareness) (Gamma = 0.659, df = 4, *p* < 0.001). Table 1 shows a cross tabulation between respondents’ year of study and their understanding of the permission of the slaughter of animals without stunning under UK animal welfare regulations.

In a separate question, respondents were asked to share their opinion on the use of pre-slaughter stunning during meat production; 95.2% (437) of respondents indicated that all animals must be stunned before slaughter, including during religious slaughter, 2.2% (10) selected ‘other’ with the option to leave comments (Table 2 shows the comments left by these 10 respondents), 1.5% (7) indicated that they did not have an opinion on stunning and 1.1% (5) indicated that religious slaughter should be exempt from stunning to comply with traditional religious values. Respondents were also asked for their views on whether there is a need for meat to be labelled according to the method of slaughter (i.e., whether meat is from an animal that has been stunned or not). A total of 97.2% (446) indicated that meat should be labelled according to whether it was derived from stunned or nonstun animals, 1.3% (6) indicated that there is no need to label meat, whilst 1.5% (7) indicated they did not have an opinion. To gauge respondents’ acceptability of meat derived from animals that had been effectively stunned during Halal slaughter, respondents were asked: ‘If animals are effectively stunned before Halal slaughter, as an ordinary consumer, would you wittingly purchase and consume this type of Halal meat?’. Two separate analysis were made, first, with all respondents (including vegans and vegetarians) and a second, excluding vegans and vegetarians. In the first analysis, one respondent did not answer the question, 79.0% (362) answered ‘Yes’ and 21% (96) indicated ‘No’. The results showed a significant trend with respondents’ year of study and their willingness to purchase and consume Halal meat from effectively stunned animals (Gamma = 0.210, df = 4, *p* = 0.009), an increasing proportion answering ‘yes’, with increasing year of study (Table 3). In the second analysis, which excluded vegans and vegetarians, the majority of respondents, 88.4% (327) indicated that they would wittingly purchase Halal meat from stunned animals, whilst 11.6% (43) indicated that they would not purchase Halal meat from stunned animals. This still indicated a trend of increased acceptance of Halal meat from stunned animals as respondents progressed in their years of study, but this time, the trend was lesser and not statistically significant (Gamma = 0.147, df = 4, *p* = 0.203). Data were then further analysed to examine the attitudes of vegetarians and vegans alone towards Halal meat. The results showed a significant increase in the percentage of vegetarians and vegans who would wittingly purchase Halal meat from stunned animals as year of study increased (Gamma = 0.311, df = 4, *p* = 0.042).

## 4. Discussion

The results of this study give an insight into the perception and understanding of religious slaughter issues by veterinary students at various levels of their studies from four universities in England. The results suggest some lack of a clear understanding of Halal slaughter with regard to the regulations and animal welfare issues surrounding the two main methods of slaughter, stun and nonstun. An understanding of these issues does appear to improve as they progress in their studies. The importance of the role of an independent, official veterinary surgeon (OV) in protecting animal welfare and public health cannot be underestimated. Due to the significance of their role, they require a better understanding of the burning issues around slaughter, particularly religious slaughter. The slaughter of animals under religious rites continues to attract public interest because of the insistence by a section of the religious communities that animals be slaughtered by severance of major blood vessels whilst they are fully conscious. There is scientific evidence to suggest that the slaughter of animals without stunning is painful [12,23], and loss of consciousness may be protracted [24], especially in the case of cattle were the vertebral artery is able to maintain blood supply to the brain [25]. However, a minority of the respondents in this study did not agree that stunning was necessary, by indicating that they believed ‘stunning of animals prior to slaughter cannot abolish the pain associated with the neck-cut during slaughter’. The majority of the respondents 90.4% (413), however, indicated that they understood the slaughter of animals without stunning to be painful and that stunning is capable of abolishing the pain associated with neck-cutting. There was a trend of increased awareness of respondents’ responses to whether stunning is capable of abolishing the pain associated with the neck-cut. This suggests that first-year students may not have yet undertaken any lectures on the science of stunning and slaughter and the rest of the years may have varying degrees of teaching, understanding and retention of the concept of stunning. Main [26] suggested that variation in veterinary curriculum and the way veterinary students are taught may account to variation in the level of understanding of students in the ever-evolving animal welfare module.

On the situation with regard to stunning of animals before Halal slaughter, the responses generally showed some lack of understanding of the facts surrounding Halal slaughter in the UK and suggest that this material needs to be better presented to students. This corroborates the conclusion made by Main [26], who noted that there is a need for veterinary institutions to include some core components of animal welfare in their curriculum in order to offer students a better appreciation of welfare science, ethics and standards. There is sufficient evidence from animal welfare surveys to show that the majority of animals are stunned during Halal slaughter in the England and Wales [27] and also within the EU [28]. There were, however, 23.1% (106) of respondents who indicated that they thought the majority of animals are stunned before Halal slaughter; this is consistent with the current situation in the England and Wales and parts of Europe [27,28]. Respondents’ understanding on this issue (situation with regard to stunning of animals before Halal slaughter) tends to improve in later years of their study, with students describing the situation in line with current practices. On the acceptability to veterinary students of Halal meat from animals that have been stunned, the majority of respondents indicated that they would buy and consume Halal meat from stunned animals. Respondents who indicated that they would avoid stunned Halal meat may have done so with the belief that Halal stunning is not as humane as conventional stunning, or they may have done so for reasons not related to animal welfare. Levine and colleagues [29] observed differences in the level of understanding of humane procedures between US veterinary students with aspirations to work with food animals from those aspiring to work with companion animals; this may account for why some students consciously avoided meat derived from animals stunned with stunning methods they may perceive to be inhumane. Similarly, Mariti and others [30] observed that veterinary students in Italy gave more consideration to the welfare of companion animals than that of food animals; this may affect their perception and understanding of animal welfare issues around food animals. It must be noted that there is no real procedural difference between stunned Halal and conventional slaughter (with the exception of a short prayer during Halal slaughter). Therefore, the humaneness of stunned Halal slaughter is not inferior to the humaneness of conventional slaughter, and one cannot therefore use humaneness (or the lack of it) as a reason to avoid meat from effectively stunned Halal slaughter. Interestingly, the results also showed a significant increase in the percentage of vegans and vegetarians with year of study who would wittingly buy Halal meat from stunned animals. Although, presumably, vegans and vegetarians will usually avoid purchasing meat, their responses may have been for a number of reasons: (i) Some respondents may have been vegetarians who would consume meat if the humaneness of slaughter was guaranteed, (ii) the level of understanding of vegetarians and vegans on animal welfare issues (particularly humane slaughter) may have improved as they progressed in the level of study or (iii) they may just have given hypothetical answers to the question as they were only given the ‘yes’ or ‘no’ alternatives and did not feel they could miss a section of the questionnaire. In retrospect, more thought should have gone into the questionnaire to avoid ambiguity in interpretation of the responses made by the vegetarians and vegans to this question. Beardsworth and Keil [31], in a study on vegan and vegetarian trends, reported that ethics and welfare were the main reasons why some consumers avoided meat. One may argue that if some vegans and vegetarians can be assured of the highest welfare of animals, it may change their consumption pattern, which may explain this finding.

In line with scientific opinion on the welfare aspects of slaughter without stunning [12,13,14], the majority of respondents indicated that all animals must be stunned before slaughter. This is consistent with the observation made by Broom [32], who reported that there is increased awareness around animal welfare at slaughter which has led to an increasing number of consumers demanding humanely slaughtered products or avoiding those associated with poor welfare. A small proportion of respondents indicated that they did not have an opinion on stunning. With the recent rise in campaigning for restrictions or a ban on nonstun slaughter by the veterinary profession and other animal welfare charities (e.g., the British Veterinary Association, Royal Society for the Prevention of Cruelty to Animals), one would expect veterinary students to be well informed and hold an opinion on pre-slaughter stunning. The issue is further highlighted by the ten respondents who selected ‘other’ and left comments to support their choice (see Table 2). The comments showed variation in the opinion of veterinary students with regard to stunning and slaughter; some respondents questioned the humaneness of stunning and others called for the current exemption of religious slaughter from stunning to be withdrawn. One respondent cited their cultural upbringing as a factor that conflicts with their profession and opinion on stunning; the influence of the culture of students on their perception of animal welfare issues has been discussed by Philip and McCulloch [33], who reported that students’ attitudes to animal welfare were influenced by their cultural upbringing, with students from Europe and the USA less likely to ‘condone cruelty to animals’. A minority of respondents indicated that in their opinion, religious slaughter should be exempt from stunning to comply with traditional religious values. This is the case in some but not all EU member states (e.g., the UK, France, Germany and others), where a derogation is applied to permit the slaughter of animals without stunning for religious rites.

The results showed a higher proportion of vegans, 3.9% (18), compared with the general UK population, which is estimated at around 1.16% [34]. This may be in part due to an increased empathy for animals by veterinary students due to their close association and everyday contact with animals. A minority of the respondents (less than 1%) indicated that they did not eat meat due to ‘dietary exclusion by choice. These respondents neither identified themselves as vegans, vegetarians, nor meat eaters. The assumption is that they probably had an intolerance, allergic or medical reason for not consuming meat.

## 5. Conclusions

As future enforcers of the law at the time of slaughter (including Halal), it is important for veterinary institutions in the UK to introduce students to the science and politics surrounding religious slaughter at all stages of their veterinary education so that students will be well informed about these issues on qualifying. It appears that for the majority of veterinary students, the debate surrounding Halal meat production is not concerned with religious ideas, but animal welfare, with the majority of respondents indicating that they would consciously consume Halal meat if it was obtained from animals that have been effectively stunned. Vegetarianism and veganism are slightly increased among veterinary students in comparison with the general UK population. It is recommended that future studies on this topic should consider evaluating the curriculum of different universities to examine whether there are disparities in teaching on religious slaughter, and how it might be improved in general.

## Figures and Tables

**Table 1:** Cross tabulation of the year of study of respondent and awareness that slaughter without stunning for religious purposes was permissible in the UK.

Year of Study	UK Welfare Regulations Do Permit the Slaughter of Animals without Stunning, but Only for Religious Slaughter. Were You Aware of This?		
	No	Yes	Total
1	29% (20)	71% (48)	100% (68)
2	14% (14)	86% (83)	100% (97)
3	5% (5)	95% (99)	100% (104)
4	4% (4)	96% (101)	100% (105)
5	1% (1)	99% (83)	100% (84)
Totals	44	414	(458)

**Table 2:** Comments by respondents who choose the option ‘other’ to the question regarding their opinion on the use of pre-slaughter stunning for meat animals.

There is no such thing as humane slaughter, no animal wants to die, therefore it is not humane
I do not agree with the exemption of stunning pre-slaughter and believe that stunning should be performed. This being said, I respect the choices and beliefs of religions other than my own.
All animals should be stunned before slaughter if is the most humane way—it is insanity that religion could come before the welfare of sentient beings.
I think a method should be employed to ensure the animals can’t feel the pain of the true cause of death. I was told by a veterinarian that stunning renders the animal essentially ‘brain-dead’ and so prevents the feeling of pain during slaughter—any other method that achieves the same effect without causing further pain or discomfort to the animal would also be appropriate.
Conflicting between the two I have an opinion, but it changes whether I draw from my cultural upbringing or my veterinary knowledge.
Animals should not be slaughtered for meat at all, however whilst this continues to happen, they should all be stunned including for religious slaughter.
If animals aren’t stunned, meat in stores should be labelled accordingly so that people can be informed/choose not to eat non-stunned meat.
I think it is difficult as people want to follow what their religion says, and I think they have that right, but I also think the welfare of the animal is important so I’m not sure if religious slaughter should be exempt from stunning or not.
Stunning makes us feel better at the animal doesn’t display a classic pain response. It’s impossible for us to actually know the level of pain the animal feels after stunning. If stunning does eliminate the pain, then it should be done in all cases of slaughter regardless of religious beliefs.
I agree most closely with the first option however I would rather non recoverable stun / killing methods were used to eliminate pain from recovery.

**Table 3:** Cross tabulation of the year of study of respondent and willingness to buy and consume Halal meat derived from animals that have been stunned before slaughter.

Year of Study	If Animals Are Effectively Stunned before Halal Slaughter, as an Ordinary Consumer, Would You Wittingly Purchase and Consume This Type of Halal Meat?		
	Yes	No	Total
1	69% (47)	31% (21)	100% (68)
2	75% (73)	25% (24)	100% (97)
3	82% (85)	18% (19)	100% (104)
4	81% (85)	19% (20)	100% (105)
5	86% (72)	14% (12)	100% (84)
Total	362	96	458
